# Alpha-Fetoprotein Ratio Predicts Alpha-Fetoprotein Positive Hepatocellular Cancer Patient Prognosis after Hepatectomy

**DOI:** 10.1155/2022/7640560

**Published:** 2022-01-11

**Authors:** Li-Yue Sun, Wen-Jian Cen, Wen-Ting Tang, Ling Deng, Fang Wang, Xiao-Meng Ji, Jiao-Jiao Yang, Ren-Jing Zhang, Xu-Hui Zhang, Zi-Ming Du

**Affiliations:** ^1^Second Department of Oncology, Guangdong Second Provincial General Hospital, 466 Xingang-Zhong Road, Guangzhou, China; ^2^State Key Laboratory of Oncology in South China, Guangzhou, China; ^3^Collaborative Innovation Center for Cancer Medicine, Guangzhou, China; ^4^Department of Molecular Diagnostics, Sun Yat-sen University Cancer Center, Guangzhou, China

## Abstract

**Background:**

This study was conducted to investigate the effect of alpha-fetoprotein (AFP) ratio on the prognosis of AFP-positive hepatocellular carcinoma (HCC) patients after hepatectomy.

**Methods:**

We retrospectively included 879 HCC patients with AFP-positive who underwent hepatectomy from February 2012 to October 2017 and randomly divided into training cohort and validation cohort. AFP ratio was equal to the AFP level within one week before hepatectomy to AFP level within 20-40 days after surgery. The end point of follow-up was disease-free survival (DFS) and overall survival (OS).

**Results:**

AFP ratio was not associated with clinical characteristics in training cohort and validation cohort. According to the X-tile software, the optimum cut-off point was 17.8 for AFP ratio. Significant differences between AFP ratio high and AFP ratio low were observed in DFS and OS in both cohort (*p* < 0.05). Kaplan-Meier curves and receiver-operating curves were showed that AFP ratio was better than AFP level preoperation in predicting the prognosis of AFP-positive HCC patients after hepatectomy. The multivariate analysis demonstrated that AFP ratio was a significant independent risk factor for both OS and DFS in HCC patients with AFP-positive.

**Conclusions:**

AFP ratio might be a prognosis predictor for HCC patients with AFP-positive after hepatectomy.

## 1. Introduction

Hepatocellular carcinoma (HCC) is a common malignancy of the digestive system in China [1]. Hepatectomy remains the most effective treatment for HCC patient without metastasis [2]. However, there existed conventional prognostic indicators that are poor at predicting the prognosis of HCC after hepatectomy [3]. Alpha-fetoprotein (AFP) has been used as an indicator of HCC diagnosis and prognosis, but only 60% of patients with HCC have positive AFP [4]. Previous studies have shown that AFP-positive was associated with worse biological behavior and inferior survival compared with AFP-negative patients [5]. However, for now there is no recognized prognostic tool available for AFP-positive HCC patients after hepatectomy.

Nobuoka et al. found that the negative/positive changes before and after hepatectomy can predict the postoperative prognosis of HCC patients [6]. Another study showed that the prognosis of AFP-positive HCC patients with a postoperative decrease of more than 50% AFP is better than that of patients with a postoperative decrease of less than 50% [7]. These studies indicate that the before or after operative change of the AFP level can be used as a predictive indicator of HCC especially AFP-positive HCC.

Therefore, in this retrospective study, we aimed at investigating the prognosis effect of AFP level before/after operation ratio (AFP ratio) in AFP-positive HCC patients after surgery.

## 2. Materials and Methods

### 2.1. Patient Enrollment

From February 2012 to October 2017, a total of 879 HCC patient undergoing resection at Sun Yat-sen University Cancer Center were selected for this retrospective analysis. Inclusion criteria included the following: (1) diagnosed with HCC confirmed by pathological examination; (2) hepatectomy as first-line treatment; (3) R0 resection; (4) preoperative serum AFP level was positive; (5) all patients had a well-documented clinical history and detailed follow-up information. The exclusion criteria included second primary tumor and distant metastasis. All patients were regrouped according to the 8th American Joint Committee on Cancer (AJCC) tumor-node-metastasis (TNM) staging system. Ethics approval was given by the institution ethics committee (approval number B2019-019-01).

### 2.2. AFP Ratio Calculated

The AFP ratio was defined as the ratio of AFP values within one week before hepatectomy to AFP values within 20-40 days after surgery. The normal range of AFP in the hospital is 0-25 ng/*μ*l.

### 2.3. Follow-Up

This study endpoints were overall survival (OS) and disease-free survival (DFS). The subsequent follow-up after surgery was conducted according to the National Comprehensive Cancer Network guidelines, including serum AFP levels, enhanced computed tomography (CT), or enhanced magnetic resonance imaging (MRI) in every 3 months during the first 2 years after hepatectomy and every 6 months thereafter. Recurrence is according to enhanced CT, MRI, or medical record.

### 2.4. Statistical Analysis

Statistical analysis was performed using SPSS software (SPSS version 20.0, SPSS). Continuous data were analyzed using the *t*-test and one-way analysis of variance (ANOVA). Categorical data were tested using the chi-square test. The ROC curve was used to evaluate the prognostic accuracy of the AFP ratio and AFP value before hepatectomy. Survival curves were estimated by the Kaplan–Meier (KM) method. A 2-tail *p* value below 0.05 are considered significant.

## 3. Results

### 3.1. Clinical Characteristics of AFP-Positive HCC Patients

The median follow-up time was 25.57 months. Patients were grouped into a training cohort (*n* = 439) and a validation cohort (*n* = 440) with random number method [8]. The results showed that there were no statistically significant differences in the clinical characteristics between the two cohorts (see Table 1).

### 3.2. Association between AFP Ratio and Clinical Characteristics of AFP-Positive HCC Patients

We used X-tile software to determine the AFP ratio cut-off value (17.8) in training cohort [9]. In the training cohort, the ALBI score of patients with AFP ratio > 17.8 (AFP ratio high) was lower than patients with AFP ratio ≤ 17.8 (AFP ratio low) (AFP ratio high vs. AFP ratio low, −2.99 ± 0.30 vs. −2.89 ± 0.34, *p* = 0.003). In the validation cohort, the patients with or without cirrhosis had a significant difference between AFP ratio high and AFP ratio low (*p* = 0.009). The AFP before hepatectomy of AFP ratio high was higher than AFP ratio low and the AFP after hepatectomy of AFP ratio high was lower than AFP ratio low, as we had expected. The other clinical and laboratory parameters did not vary between AFP ratio high and AFP ratio low in the two cohorts (see Table 2).

### 3.3. Prognostic Value of AFP Ratio in Patients with AFP-Positive HCC

**Figure 1:**
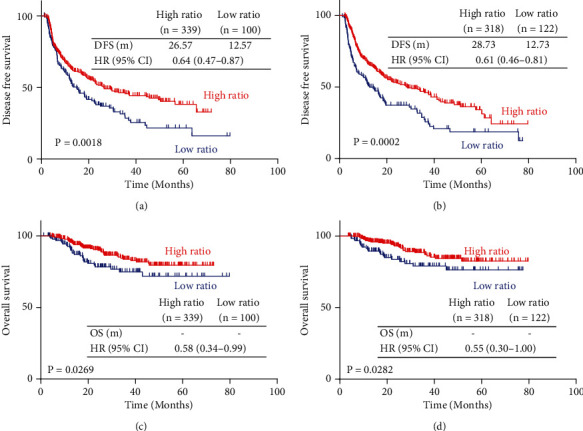
Kaplan-Meier curves showing the DFS of AFP ratio high and AFP ratio low HCC patients with AFP-positive in the training cohort (a) and validation cohort (b). Kaplan-Meier curves showing the OS of AFP ratio high and AFP ratio low HCC patients with AFP-positive in the training cohort (c) and validation cohort (d). AFP: alpha-fetoprotein; HCC: hepatocellular carcinoma; DFS: disease-free survival; OS: overall survival.

We next investigated the prognostic value of AFP ratio in AFP-positive HCC patients after resection. In the training cohort, the DFS (26.57 months with AFP ratio high vs. 12.57 months with AFP ratio low, *p* = 0.0018; HR, 0.64, 95% CI, 0.47-0.87) and OS (*p* = 0.0269; HR, 0.58, 95% CI, 0.34-0.99) of AFP ratio high patients was significantly longer than AFP ratio low patients. In the validation cohort, the DFS (28.73 months with AFP ratio high vs. 12.73 months with AFP ratio low, *p* = 0.0020; HR, 0.61, 95% CI, 0.46-0.81) and OS (*p* = 0.0282; HR, 0.55, 95% CI, 0.30-1.00) of AFP ratio high patients was significantly longer than for those that were AFP ratio low patients (see Figure 1).

We further analyzed the predictive value of AFP ratio in early HCC recurrence and later HCC recurrence in the whole patients. According to previous study, early recurrence was defined as recurrence within 1 year after surgery and later recurrence was defined as recurrence after 1 year [10]. The results showed no statistically significant difference of AFP ratio between early HCC recurrence patients and later HCC recurrence patients (early recurrence vs. later recurrence, 78.06 ± 8.23 vs. 90.78 ± 14.42, *p* = 0.41) (see Supplementary Figure (available here)). The proportion of AFP ratio high among early recurrence was similar to that among later recurrence (AFP ratio high in early recurrence was 64.55% (213/330); AFP ratio high in later recurrence was 64.91% (111/171)).

### 3.4. AFP Level in the Prognostic Value of HCC Patients

**Figure 2:**
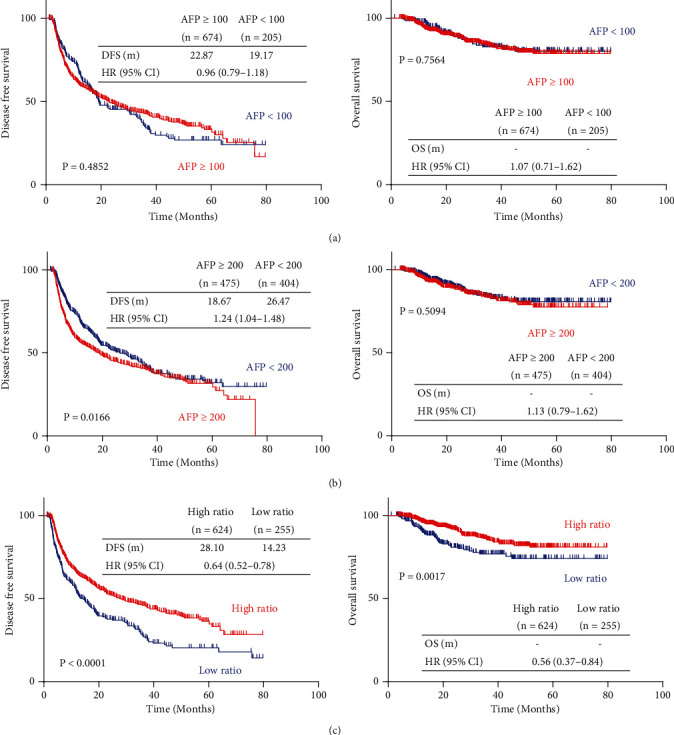
Kaplan-Meier curves showing the DFS and OS of preoperation AFP level ≥ 100 ng/*μ*l (a), preoperation AFP level ≥ 200 ng/*μ*l (b), and AFP ratio (c) in AFP-positive HCC patients. AFP: alpha-fetoprotein; HCC: hepatocellular carcinoma; DFS: disease-free survival; OS: overall survival.

AFP ≥ 100 ng/*μ*l or AFP ≥ 200 ng/*μ*l is generally considered to be a useful indicator for HCC patients prognostic after surgery [11, 12]. We compared AFP ratio and AFP level in predicting prognosis of AFP-positive HCC patients after surgery. The results showed that no difference in DFS or OS was found between AFP ≥ 100 ng/*μ*l patients and AFP < 100 ng/*μ*l patients (DFS, AFP ≥ 100 ng/*μ*l vs. AFP < 100 ng/*μ*l, 22.87 vs. 19.17 months, *p* = 0.4852; HR, 0.96, 95% CI, 0.79-1.18; OS, *p* = 0.7564; HR, 1.07, 95% CI, 0.71-1.62). AFP ≥ 200 ng/*μ*l can predict the DFS of AFP-positive HCC patients after hepatectomy (AFP ≥ 200 ng/*μ*l vs. AFP < 200 ng/*μ*l, 18.67 vs. 26.47 months, *p* = 0.0166; HR, 1.24, 95% CI, 1.04-1.48), but not OS (*p* = 0.5094; HR, 1.13, 95% CI, 0.79-1.62). The DFS of HCC patients with high AFP ratio was significantly longer than that of patients with low AFP ratio (AFP ratio high vs. AFP ratio low, 28.10 vs. 14.23 months, *p* < 0.0001; HR, 0.64, 95% CI, 0.52-0.78), and OS was also significantly prolonged (*p* = 0.0017; HR, 0.56, 95% CI, 0.37-0.84) (Figure 2).

**Figure 3:**
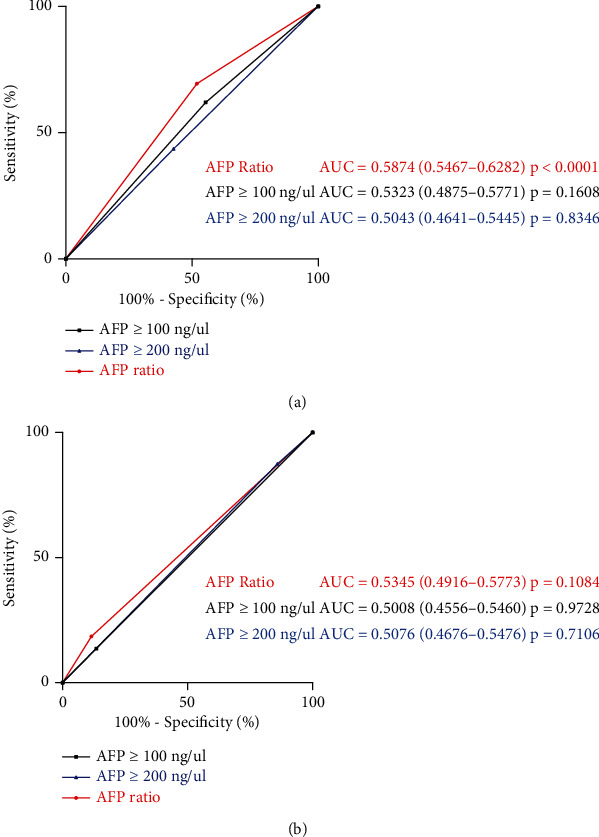
Comparison of ROC curves of preoperation AFP level ≥ 100 ng/*μ*l, preoperation AFP level ≥ 200 ng/*μ*l, and AFP ratio in prognostic evaluation of DFS (a) and OS (b) in AFP-positive HCC patients. AFP: alpha-fetoprotein; HCC: hepatocellular carcinoma; DFS: disease-free survival; OS: overall survival.

We used ROC curve to compare the prognostic efficacy of AFP ratio, AFP ≥ 100 ng/*μ*l, and AFP ≥ 200 ng/*μ*l. With an AUC of 0.5875 (95% CI, 0.5467-0.6282) on prediction of recurrence, AFP ratio outperformed AFP level (AFP ≥ 100 ng/*μ*l, AUC = 0.5323 (95% CI, 0.4875-0.5771); AFP ≥ 200 ng/*μ*l, AUC = 0.5043 (95% CI, 0.4641-0.5445)). In OS prediction, the AUC of the AFP ratio was 0.5345 (95% CI, 0.4916-0.5773), which was similar to AFP level (AFP ≥ 100 ng/*μ*l, AUC = 0.5008 (95% CI, 0.4556-0.5460); AFP ≥ 200 ng/*μ*l, AUC = 0.5076 (95% CI, 0.4676-0.5476)) (Figure 3).

### 3.5. Univariate and Multivariate Survival Analyses in Patients with AFP-Positive HCC

The results of the univariate and multivariate analysis were listed in Tables 3 and 4. Multivariate analysis indicated that AFP ratio > 17.8 was an independent predictor of DFS and OS of AFP-positive HCC patients (DFS, HR, 1.710, 95% CI, 1.422-2.056, *p* < 0.001; OS, HR, 2.004, 95% CI, 1.381-2.906, *p* < 0.001). The results showed that tumor size > 5 cm, microscopic vascular invasion, macroscopic vascular invasion, and tumor multiplicity were also independent poor prognostic factors for AFP-positive HCC patients regarding DFS but not OS (Tables 3 and 4).

## 4. Discussion

Clinically, AFP-positive HCC patients presented high malignancy, rapid progression and poor prognosis compared to AFP-negative HCC patients [13]. Previous research showed AFP-positive HCC patients with 5-year survival rate of 26.7% compared to AFP-negative HCC patients with 5-year survival rate of 56.5% [14]. However, there was lack of efficient prognostic indicators of AFP-positive HCC patients after surgery. In this study, we explored the role of AFP ratio in AFP-positive HCC patients after surgery by conducting a retrospective analysis.

AFP is wildly used for diagnosis, prognosis, and surveillance of HCC [15]. According to the level of serum AFP that can be used clinically, we generally divide the HCC patients to AFP-positive and AFP-negative [16]. HCC patients with AFP-positive and AFP-negative showed significant differences in clinical. Prior studies have suggested the association between high AFP levels and poor outcome of patients with HCC and some of staging systems like the Cancer of the Liver Italian Pro-gram score (CLIP score) [17] and Biomarker combined Japan Integrated Staging (bm-JIS) [18] already incorporated the level of serum AFP [19]. Therefore, only few studies focused on prognostic biomarkers in AFP-positive HCC; others mainly focused on AFP-negative HCC or overall HCC patients.

 Toro et al. found AFP level of pre- and posttreatment correlated with survival of HCC patients [20]. Nobuoka et al. also showed that the positive and negative changes of AFP before and after hepatectomy can also predict the postoperative recurrence of HCC [6]. Another study showed that AFP ratio could predict recurrence in HCC after liver transplant [21]. In the present study, we randomly separated AFP-positive HCC patients into a training cohort and validation cohort and found that HCC patients with high AFP ratio had better OS and DFS in the two cohorts. The results suggested that AFP ratio could be a potential prognostic biomarker in AFP-positive HCC patients after hepatectomy.

Multiple studies have indicated that AFP level could be used as a prognostic marker of HCC patients after hepatectomy [11, 12]. However, the effect of AFP level on AFP-positive HCC after resection is still unclear. Our study found that preoperative AFP ≥ 100 ng/*μ*l could not predict the prognosis of AFP-positive HCC patients. Preoperative AFP ≥ 200 ng/*μ*l was only related to DFS, but not OS. ROC curve also showed that AFP ratio had better prognostic diagnostic efficacy for HCC patients with AFP-positive than AFP ≥ 100 ng/*μ*l and AFP ≥ 200 ng/*μ*l. The above results suggested a limited prognostic role of AFP level before hepatectomy and AFP ratio could be used as a better prognostic indicator for AFP-positive HCC patients.

Univariate and multivariate analysis also showed that AFP ratio was an independent risk predictor in DFS and OS in AFP-positive HCC patients. Previous studies have identified several prognostic indicators for HCC patients after hepatectomy, including tumor size [22], TNM stage [22], and vascular invasion [23]. However, these indicators were found no significant difference in OS in this study. Therefore, AFP ratio is a prognosis predictor of HCC patients with AFP-positive.

 Our study showed AFP ratio as a prognostic marker in AFP-positive HCC after surgery. However, there were several shortcomings in this study: (1) there might be a risk of bias in the study due to one single center retrospective study; (2) the AFP level was quantified by different approaches, and the AFP ratio used might need more validation.

## 5. Conclusions

This study retrospectively analyzed AFP-positive HCC patients undergoing hepatectomy in a large cohort. The AFP ratio showed the prognostic significance of AFP-positive HCC patients after surgery, which was validated in both training and validation sets. AFP ratio showed better prognostic predictive value than AFP levels in AFP-positive HCC patients. This study provided a potential prognostic indicator for AFP-positive HCC patients.

## Figures and Tables

**Table 1:** The clinical feature of patients in training cohort and validation cohort.

	Training cohort	Validation cohort	*p* value
Number	439	440	
Age (year)	49.46 ± 11.61	50.11 ± 12.45	0.420
Gender			
Male	374 (85.2)	387 (88.0)	0.230
Female	65 (14.8)	53 (12.0)	
Smoking history, *n* (%)			
Yes	193 (44.0)	203 (46.1)	0.792
No	246 (56.0)	237 (53.9)	
Alcohol history, *n* (%)			
Yes	93 (21.2)	109 (24.8)	0.206
No	346 (78.8)	331 (75.2)	
HBsAg, *n* (%)			
Positive	380 (86.6)	371 (84.3)	0.346
Negative	59 (13.4)	69 (15.7)	
Cirrhosis, *n* (%)			
Yes	426 (97.0)	433 (98.4)	0.173
No	13 (3.0)	7 (1.6)	
Child-Pugh score	5.06 ± 0.31	5.04 ± 0.23	0.321
Child-Pugh class			
A	431 (98.2)	437 (99.3)	0.128
B	8 (1.8)	3 (0.7)	
MELD score	4.22 ± 2.80	4.45 ± 2.65	0.207
ALBI score	−2.96 ± 0.32	−2.93 ± 0.32	0.221
TNM stage, *n* (%)			0.505
IA	53 (12.1)	38 (8.6)	
IB	149 (33.4)	155 (35.2)	
II	160 (36.4)	160 (36.4)	
IIIA	34 (7.7)	36 (8.2)	
IIIB	43 (9.8)	51 (11.6)	
Tumor size (cm)	5.53 ± 3.64	5.54 ± 3.39	0.950
Differentiation, *n* (%)			0.869
Well-moderate	186 (42.4)	184 (41.8)	
Poor-undifferentiated	253 (57.6)	256 (58.2)	
Microscopic vascular invasion, *n* (%)			0.289
Yes	182 (41.5)	198 (45.0)	
No	257 (58.5)	242 (55.0)	
Macroscopic vascular invasion, *n* (%)			0.740
Yes	41 (9.3)	44 (10.0)	
No	388 (90.7)	396 (90.0)	
Tumor multiplicity, *n* (%)			0.296
Single	349 (79.5)	362 (82.3)	
Multiple	90 (20.5)	78 (17.7)	
AFP before hepatectomy (ng/*μ*l)	12889.34 ± 33411.38	9818.80 ± 25108.39	0.124
AFP after hepatectomy (ng/*μ*l)	303.41 ± 1148.90	275.51 ± 1458.27	0.753
AFP ratio	90.97 ± 212.37	92.68 ± 160.20	0.892

**Table 2:** The clinical feature of patients with AFP ratio high and AFP ratio low in 2 cohort.

	Training cohort		*p* value	Validation cohort		*p* value
	AFP ratio high	AFP ratio low		AFP ratio high	AFP ratio low	
Number	306	133		318	122	
Age	49.15 ± 12.00	50.16 ± 10.68	0.406	50.04 ± 12.46	50.30 ± 12.48	0.124
Gender						
Male	254 (83.0)	120 (90.2)	0.050	275 (86.5)	112 (91.8)	0.051
Female	52 (17.0)	13 (9.8)		43 (13.5)	10 (8.2)	
Smoking history, *n* (%)						
Yes	131 (42.8)	62 (46.6)	0.460	148 (46.5)	55 (45.1)	0.783
No	175 (57.2)	71 (53.4)		170 (53.5)	67 (54.9)	
Alcohol history, *n* (%)						
Yes	60 (19.6)	33 (24.8)	0.220	80 (25.2)	29 (23.8)	0.763
No	246 (80.4)	100 (75.2)		238 (74.8)	93 (76.2)	
HBsAg, *n* (%)						
Positive	266 (86.9)	114 (85.7)	0.732	262 (82.4)	109 (89.3)	0.073
Negative	40 (13.1)	19 (14.3)		56 (17.6)	13 (10.7)	
Cirrhosis, *n* (%)						
Yes	298 (97.4)	128 (96.2)	0.515	316 (99.4)	117 (95.9)	**0.009**
No	8 (2.6)	5 (3.8)		2 (0.6)	5 (4.1)	
Child-Pugh score	5.05 ± 0.26	5.10 ± 0.39	0.103	5.03 ± 0.19	5.07 ± 0.32	0.090
Class						
A	302 (98.7)	129 (97.0)	0.221	317 (99.7)	120 (98.4)	0.131
B	4 (1.3)	4 (3.0)		1 (0.3)	2 (1.6)	
MELD score	4.11 ± 2.56	4.47 ± 3.27	0.214	4.26 ± 2.65	4.96 ± 2.61	0.013
ALBI score	−2.99 ± 0.30	−2.89 ± 0.34	**0.003**	−2.95 ± 0.31	−2.89 ± 0.33	0.056
TNM stage, *n* (%)						
IA	40 (13.1)	13 (9.8)	0.319	30 (9.4)	8 (6.6)	0.334
IB	102 (33.3)	47 (35.3)		118 (37.1)	37 (30.3)	
II	106 (34.6)	54 (40.6)		110 (34.6)	50 (41.0)	
IIIA	23 (7.5)	11 (8.3)		27 (8.5)	9 (7.4)	
IIIB	35 (11.4)	8 (6.0)		33 (10.4)	18 (14.8)	
Tumor size (cm)	5.61 ± 3.54	5.34 ± 3.86	0.470	5.62 ± 3.34	5.35 ± 3.51	0.459
Differentiation, *n* (%)						
Well-moderate	173 (56.5)	80 (60.2)	0.481	187 (58.8)	69 (56.6)	0.669
Poor-undifferentiated	133 (43.5)	53 (39.8)		131 (41.2)	53 (43.3)	
Microscopic vascular invasion, *n* (%)						
Yes	129 (42.2)	53 (39.8)	0.652	140 (44.0)	58 (47.5)	0.507
No	177 (57.8)	80 (60.2)		178 (56.0)	64 (52.5)	
Macroscopic vascular invasion, *n* (%)						
Yes	33 (10.8)	8 (6.0)	0.115	29 (9.1)	15 (12.3)	0.320
No	273 (89.2)	125 (94.0)		289 (90.9)	107 (87.7)	
Tumor multiplicity, *n* (%)						
Single	249 (81.4)	100 (75.2)	0.140	264 (83.0)	98 (80.3)	0.508
Multiple	57 (18.6)	33 (24.8)		54 (17.0)	24 (19.7)	
AFP before hepatectomy (ng/*μ*l)	16564.39 ± 37311.78	4433.95 ± 19597.33	**<0.001**	12099.86 ± 26777.70	3873.06 ± 18958.05	**0.002**
AFP after hepatectomy (ng/*μ*l)	227.66 ± 667.27	477.70 ± 1818.57	**0.036**	181.74 ± 629.40	519.92 ± 2567.91	**0.029**

**Table 3:** Univariate and multivariate analyses of the disease-free survival of AFP-positive HCC patients.

	Univariate		Multivariate	
	HR (95% CI)	*p* value	HR (95% CI)	*p* value
Gender (male)	1.467 (1.100-1.955)	**0.009**	1.231 (0.921-1.646)	0.160
Age < 50 years	1.168 (0.980-1.392)	0.083		
Smoking	1.134 (0.951-1.352)	0.161		
Drinking	1.216 (0.994-1.487)	0.057		
HBV infected	0.885 (0.692-1.131)	0.330		
Cirrhosis	1.146 (0.646-2.032)	0.641		
Child-Pugh A	0.777 (0.368-1.639)	0.508		
TNM stage III	2.397 (1.959-2.933)	**<0.001**	0.779 (0.549-1.105)	0.162
Tumor size > 5 cm	1.885 (1.581-2.247)	**<0.001**	0.697 (0.553-0.878)	**0.002**
Tumor size > 3 cm	1.785 (1.458-2.185)	**<0.001**	0.828 (0.644-1.065)	0.141
Differentiation poor	1.229 (1.027-1.469)	**0.024**	1.110 (0.927-1.330)	0.257
Microscopic vascular invasion	1.876 (1.573-2.238)	**<0.001**	1.472 (1.220-1.776)	**<0.001**
Macroscopic vascular invasion	2.316 (1.788-3.000)	**<0.001**	1.537 (1.055-2.240)	**0.025**
Tumor multiplicity	1.730 (1.411-2.121)	**<0.001**	1.325 (1.029-1.706)	**0.029**
AFP > 100 ng/*μ*l	0.964 (0.788-1.180)	0.725		
AFP > 200 ng/*μ*l	1.104 (0.918-1.328)	0.295		
AFP ratio < 17.8	1.572 (1.308-1.888)	**<0.001**	1.710 (1.422-2.056)	**<0.001**

**Table 4:** Univariate and multivariate analyses of the overall survival of AFP-positive HCC patients.

	Univariate		Multivariate	
	HR (95% CI)	*p* value	HR (95% CI)	*p* value
Gender (male)	1.343 (0.740-2.439)	0.332		
Age < 50 year	0.976 (0.681-1.398)	0.894		
Smoking	1.134 (0.951-1.352)	0.654		
Drinking	1.655 (1.131-2.421)	**0.009**	1.467 (0.998-2.155)	0.051
HBV infected	0.896 (0.543-1.480)	0.668		
Cirrhosis	0.817 (0.302-2.216)	0.692		
Child-Pugh A	20.419 (0.005-75989.228)	0.472		
TNM stage III	3.015 (2.072-4.389)	**<0.001**	0.610 (0.309-1.206)	0.155
Tumor size > 5 cm	2.381 (1.648-3.439)	**<0.001**	0.679 (0.418-1.101	0.116
Tumor size > 3 cm	2.492 (1.554-3.994)	**<0.001**	0.670 (0.375-1.196)	0.176
Differentiation poor	1.489 (1.024-2.166)	**0.037**	0.667 (0.417-1.099)	0.114
Microscopic vascular invasion	1.952 (1.359-2.805)	**<0.001**	1.334 (0.902-1.974)	0.148
Macroscopic vascular invasion	2.699 (1.697-4.294)	**<0.001**	0.598 (0.306-1.169)	0.133
Tumor multiplicity	1.771 (1.185-2.647)	**0.005**	1.251 (0.738-2.120)	0.407
AFP > 100 ng/*μ*l	1.069 (0.700-1.634)	0.756		
AFP > 200 ng/*μ*l	1.176 (0.800-1.729)	0.410		
AFP ratio < 17.8	1.785 (1.236-2.578)	**0.002**	2.004 (1.381-2.906)	**<0.001**

## Data Availability

The data used to support the findings of this study are available from the corresponding author upon request.

## Supplementary Materials

Supplementary Materials Supplementary Figure: Comparison of AFP ratio in AFP-positive HCC patients with early recurrence and later recurrence.
