# Supplementary material for: Alpha-Fetoprotein Ratio Predicts Alpha-Fetoprotein Positive Hepatocellular Cancer Patient Prognosis after Hepatectomy
Source: Dis Markers. 2022 Jan 11;2022:7640560. doi: 10.1155/2022/7640560 (PMC8766187; doi:10.1155/2022/7640560)
Supplement: Supplementary Materials — Supplementary Figure: Comparison of AFP ratio in AFP-positive HCC patients with early recurrence and later recurrence. [file 7640560.f1.docx]

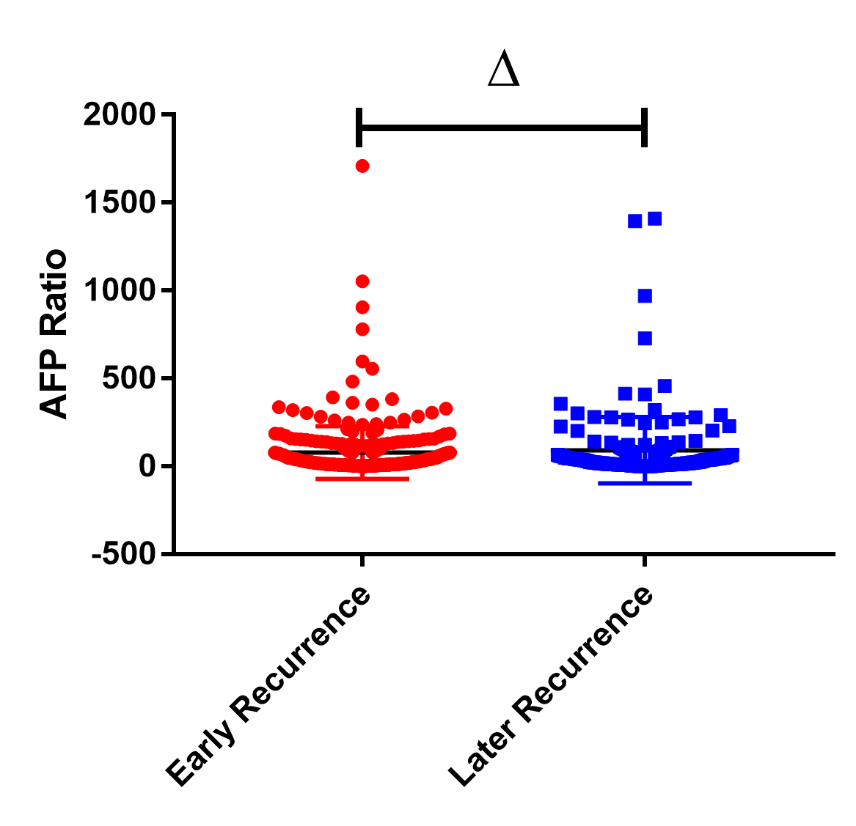


**Supplementary Figure:** Comparison of AFP ratio in AFP-positive HCC patients with early recurrence and later recurrence.

AFP, alpha-fetoprotein; HCC, hepatocellular carcinoma;
